# Measuring geographic proximity to family medicine and continuity of care in the last year of life: A population-level retrospective cohort study using Canadian health administrative data

**DOI:** 10.1371/journal.pone.0360638

**Published:** 2026-10-16

**Authors:** Shuaib Hafid, Lawrence Mbuagbaw, Bruce Newbold, Andrew Costa, Ana Gayowsky, Peter Gozdyra, Aaron Jones, Sarina R. Isenberg, Erin Gallagher, Michelle Howard

**Affiliations:** 1 Department of Family Medicine, McMaster University, Hamilton, Ontario, Canada; 2 Department of Health Research Methods, Evidence, and Impact, McMaster University, Hamilton, Ontario, Canada; 3 School of Earth, Environment, & Society, McMaster University, Hamilton, Ontario, Canada; 4 ICES McMaster, Hamilton, Ontario, Canada; 5 Centre for Integrated Care, St. Joseph’s Health System, Hamilton, Ontario, Canada; 6 ICES, Toronto, Ontario, Canada; 7 Department of Medicine, University of Ottawa, Ottawa, Ontario, Canada; 8 Bruyère Health Research Institute, Ottawa, Ontario, Canada; University of Illinois Urbana-Champaign, UNITED STATES OF AMERICA

## Abstract

**Background:**

Family physicians provide most outpatient care in patients’ last year of life, but it remains unclear how geographic distance between patients and their physicians affects continuity of care. Patients living farther from care providers may experience reduced access and more fragmented care. This study examined whether geographic proximity to a family physician is associated with relational continuity of outpatient care in the last year of life.

**Methods:**

We conducted a population‑level retrospective cohort study of adults who died in Ontario, Canada, from 2021 to 2024, using linked health administrative data. Geographic proximity was measured using quartiles of the shortest driving distance (km) between each patient’s residence and their family physician’s practice location. Relational continuity was assessed using established measures capturing the concentration of visits, dispersion of care across physicians, and the temporal regularity of visits. Associations were estimated using proportional‑odds regression models adjusted for demographic, socioeconomic, and clinical factors.

**Results:**

Among 400,499 patients, the mean age at death was 76.0 years, 52.2% were male, the mean (SD) distance to their family physician was 25.5 (92.0) km, with the highest quartile residing more than 14.2 km away from their family physician. Greater distance was associated with modestly lower relational continuity: each one-quartile increase in distance was linked to lower odds of higher continuity with the usual family physician (cOR: 0.88, 95%CI: 0.87, 0.88). Sensitivity analyses using travel time and Euclidean distance produced consistent findings.

**Discussion:**

Geographic proximity is a significant determinant of outpatient continuity near the end of life. Patients living farther from their family physicians experience more fragmented care, underscoring the need for system-level strategies to reduce geographic barriers, including improved local access to primary care and enhanced virtual-care models.

## Background

Family physicians coordinate care and facilitate specialist referrals for across the life course, with this role becoming increasingly relevant for patients with serious or life-limiting illness who often experience functional decline, increasing care needs, and transitions across care settings [1–4]. We previously identified that approximately 12 of 17 outpatient visits in the last year of life were with family physicians between 2013 and 2017 in Ontario, Canada [5]. We also identified that patients had outpatient visits with 6 unique physicians across 4 unique specialties during the last year of life. The concentration of outpatient care amongst family physicians highlights the key role family physicians play in the provision of end-of-life care. Therefore, it is important to understand how continuity of care (COC) is experienced with family physicians during this period. COC is a core dimension of healthcare and has been defined as “the degree to which a series of discrete healthcare events is experienced as coherent and connected and consistent with the patient’s medical needs and personal context” [6,7]. Family physicians provide comprehensive, longitudinal care for a wide range of conditions and may themselves, deliver, co-manage, or coordinate palliative care services. As a result, continuity with care providers reflect the overall outpatient care experiences instead of care specific to an individual condition or service type.

Although we previously identified that patients living in rural regions may experience higher COC, geographic access to healthcare is often measured using broad system-level classifications such as rural versus urban residence. These approaches, including population center definitions [8–10] and rurality indices may capture contextual differences in healthcare delivery environments but do not directly measure the physical distance between patients and their respective care providers. More importantly, geographic proximity represents a distinct patient-level dimension of access that may affect continuity independent of rurality. For instance, patients living in rural regions may have fewer providers but more concentrated care while patients in urban regions may live closer to multiple providers while experiencing more fragmented care.

There is a notable evidence gap regarding how patients’ geographic proximity to their family physicians affects their receipt of outpatient care during the end of life. Previous reviews have identified a consistent ‘distance decay’ effect, where patients residing farther from their healthcare providers experience poorer outcomes. However, these studies did not focus on EOL care nor proximity to family physicians. For example, Kelly et al.’s systematic review of 108 studies identified that only three studies measured patients’ distance to family physicians; however, all three were from the United States and unrelated to EOL care [11–13]. Similarly, most Canadian research has focused on specialist or inpatient care [14,15]. Recently, however, a study using health administrative data from Ontario, Canada, examined travel distance to rostered family physicians among approximately 10 million patients and identified that patients residing beyond 30 kilometers away had higher odds of emergency department visits, lower preventative care uptake, and poorer continuity [16]. While this study did not address EOL care specifically, it does highlight the importance of proximity in primary care and underscores the need for research in the Canadian EOL context. Despite consistent evidence of a ‘distance decay’ effect in healthcare use, the relationship between geographic proximity to family physicians and COC at the end of life remains poorly understood. Therefore, we aim to examine the association between patients’ geographic proximity to their family physicians at one year before death and their outpatient COC during the last year of life.

## Methods

### Study design, data sources and population

We conducted a population-level retrospective cohort study using linked health administrative data from ICES (formerly known as the *Institute for Clinical Evaluative Sciences*). ICES is an independent, non-profit research institute whose legal status under Ontario’s health information privacy law allows it to collect and analyze health care and demographic data without consent for health system evaluation and improvement. See S1 Appendix for a description of health administrative databases accessed through ICES. This study is reported in accordance with the Reporting of studies Conducted using Observational Routinely-collected health Data (RECORD) statement [17]. Lastly, de-identified data was accessed on November 4, 2024.

The study cohort included adults who died between January 1, 2021, and December 31, 2024, in Ontario, Canada. Individuals were excluded if they: 1) were ineligible for the Ontario Health Insurance Plan during the last two years of life; 2) had a non-Ontario postal code at death; 3) did not have contact with the healthcare system within 5 years of their death date; 4) were >105 years of age at death to exclude individuals with potentially inaccurate date of birth information; 5) lived in a long-term care home within the last year of life; 6) did not have an identifiable family physician; 7) were 18 years of age at death (to exclude paediatric outpatient visits); and 8) had missing physician practice location data required to calculate geographic proximity. We excluded individuals who lived in long-term care homes because these individuals receive care within a healthcare facility where physician services are mostly provided by the on-site physicians instead of their community-based family physicians.

### Exposure: Geographic proximity to family physicians

We measured the geographic proximity of patients to their rostered family physician at one year before death in kilometers (km), using the shortest driving distance in kilometers (km), as detailed in our published protocol [18]. Distances were calculated using the ArcGIS Network Analyst function based on road network data between the population-weighted centroid of each patient’s residential postal code and their physician’s practice location postal code. Because full residential addresses are not available in health administrative data, we approximated the patient and physician’s locations using population-weighted centroids, representing the area within a postal code where most residents live instead of its geographic center – in turn increasing the accuracy of distance estimates between postal codes [18]. Following, distances were categorized into empirical quartiles to account for the skewed distribution of travel distances and to facilitate interpretation of relative differences in geographic proximity.

### Outcomes: Continuity of care at the end of life

Our study outcomes captured patients’ outpatient relational continuity at the end of life, based on outpatient physician billings with location codes of ‘Home,’ ‘Office,’ or ‘Phone’ during the last year of life. To reflect the multidimensional nature of relational continuity, we examined three continuity indices measuring distinct but related components of outpatient care patterns. Specifically, we calculated the Usual Provider of Care (UPC) index with the family physician [19], the Relative Variance (RV) Index with the family physician [20], and the modified Bice-Boxerman (BB) index across all physicians [21,22]. These indices are commonly used in health services research [23] and together capture key dimensions of relational continuity including concentration of care, dispersion across care providers and temporal regularity of visits.

First, the UPC measures the proportion of outpatient visits with the rostered family physician in the last year of life, ranging from 0 to 1, with higher values reporting greater concentration of care with the family physician. Second, the RV index measures the variability in time intervals between sequential visits with the family physician, also ranging from 0 to 1 – with higher values reporting more evenly spaced visits. Third, the modified BB index measures the dispersion of outpatient visits across physicians within each specialty, with values ranging from 0 to 1, and higher values reporting greater concentration of care within individual providers. We categorized each index into quartiles to facilitate comparisons within and between indices. Lastly, we excluded outpatient physician billings from specialties that are not directly involved in the ongoing management and provision of outpatient care as these visits are largely episodic and may not reflect ongoing therapeutic relationships (see S2 Appendix for a list of Ontario Health Insurance Plan specialty codes excluded).

### Statistical analysis

Characteristics and outcomes were summarized using means and standard deviations for continuous variables and counts and percentages for categorical variables, stratified by quartiles of geographic proximity to their family physician. Differences in characteristics and outcomes across quartiles of geographic proximity were assessed using ANOVA for continuous variables and Pearson’s Chi-square test for categorical variables. We also calculated eta-squared (η^2^) and Cramer’s V to measure the effect sizes for continuous and categorical variables, respectively. We interpreted effect sizes using conventional thresholds, with values of 0.01, 0.06, and 0.14 representing small, medium, and large effects for η^2^ and 0.10, 0.30, and 0.50 for Cramer’s V, based on Cohen’s guidelines [24–27].

We measured the association between the exposure variable and the relational continuity of care indices using cumulative link proportional-odds ordinal regression. Cumulative odds ratios (cOR) and 95% confidence intervals (CIs) were reported per 1-quartile increase in the exposure variable. We pre-specified clinically meaningful covariates [18] which were measured at one year before death. Models adjusted for: 1) age at death; 2) sex; 3) rurality status; 4) neighbourhood-level income quintile status; 5) comorbidity status, measured using the Johns Hopkins adjusted clinical diagnostic groups (ADGs) [28]; 6) active cancer treatment status during the last year of life; 7) calendar death year to adjust for temporal health system impacts; and 8) whether the patient moved residences within the last year of life. Missing covariate data were minimal; therefore, we used complete case analyses. Sensitivity models were also estimated using quartiles of driving time (minutes) and Euclidean distance (kilometres) as alternative measures of geographic proximity. To compare model performances, we examined log-likelihood, Akaike Information Criterion, and Bayesian Information Criterion values. Statistical significance was assessed at p < 0.001 (two-tailed) due to our large sample size and model estimates were reported with 95% confidence intervals. All statistical analyses were conducted using Python [29].

### Ethics

Research projects that only use data collected by ICES are exempt from research ethics board review under section 45 of Ontario’s Personal Health Information Protection Act.

## Results

After applying study exclusion criteria, 400,499 individuals were included in the cohort for analysis (Fig 1). The mean (SD) age at death was 76.0 (15.1) years, with 52.2% being male (Table 1). The mean (SD) driving distance between patients and their rostered family physicians was 25.5 (92) kms at one year before death and 12.9% resided in rural regions. Socioeconomic status, measured using the neighbourhood-level income quintiles, was skewed towards lower income, with 26.4% and 15.6% in the lowest and highest income quintiles, respectively. Patients had a mean of 8.4 (4.6) prevalent ADGs at one year before death – with approximately 20.8% having 12 or more ADGs – and nearly one-third receiving active cancer treatments in the last year of life. Lastly, 15.6% of the cohort moved during the last year of life, likely reflecting transitions to institutional care or relocating closer to caregivers.

**Table 1:** Characteristics of adults aged ≥19 years who died in Ontario (2021–2024), stratified by quartiles of distance to their family physician.

Variable	Overall(n = 400,499)	Q1: <= 2.0 km(n = 100,125)	Q2: 2.1–5.3 km(n = 100,125)	Q3: 5.4–14.2 km(n = 100,142)	Q4: > 14.2 km(n = 100,107)	P	Effect Size
Age at death, mean (SD)	76.0 (15.1)	77.6 (14.2)	77.2 (14.5)	76.2 (14.8)	72.9 (16.4)	<0.001	η^2^ = 0.015
Age categories at death, n (%)						<0.001	V = 0.071
19–44	17,412 (4.3%)	3,070 (3.1%)	3,409 (3.4%)	3,949 (3.9%)	6,984 (7.0%)		
45–54	17,418 (4.3%)	3,486 (3.5%)	3,936 (3.9%)	4,351 (4.3%)	5,645 (5.6%)		
55–64	45,322 (11.3%)	10,136 (10.1%)	10,346 (10.3%)	11,050 (11.0%)	13,790 (13.8%)		
65–74	78,416 (19.6%)	18,602 (18.6%)	18,564 (18.5%)	19,672 (19.6%)	21,578 (21.6%)		
75–84	108,085 (27.0%)	27,725 (27.7%)	27,550 (27.5%)	27,532 (27.5%)	25,278 (25.2%)		
85–94	108,876 (27.2%)	30,137 (30.1%)	29,648 (29.6%)	27,331 (27.3%)	21,760 (21.7%)		
95+	24,970 (6.2%)	6,969 (7.0%)	6,672 (6.7%)	6,257 (6.2%)	5,072 (5.1%)		
Male, n (%)	209,106 (52.2%)	50,659 (50.6%)	51,120 (51.1%)	52,635 (52.6%)	54,692 (54.6%)	<0.001	V = 0.032
Rural status at one year before death, n (%)	51,665 (12.9%)	16,249 (16.2%)	2,833 (2.8%)	8,474 (8.5%)	24,109 (24.1%)	<0.001	V = 0.239
Neighbourhood-level income quintile at one year before death, n (%)						<0.001	V = 0.093
1 (lowest quintile)	105,935 (26.4%)	35,598 (35.5%)	27,238 (27.2%)	20,936 (20.9%)	22,163 (22.1%)		
2	88,845 (22.2%)	23,874 (23.8%)	23,470 (23.4%)	20,346 (20.3%)	21,155 (21.1%)		
3	75,422 (18.8%)	16,634 (16.6%)	18,450 (18.4%)	20,112 (20.1%)	20,226 (20.2%)		
4	67,819 (16.9%)	13,287 (13.3%)	15,970 (15.9%)	18,915 (18.9%)	19,647 (19.6%)		
5 (highest quintile)	62,355 (15.6%)	10,709 (10.7%)	14,996 (15.0%)	19,821 (19.8%)	16,829 (16.8%)		
Missing	123 (0.0%)	23 (0.0%)	1 (0.0%)	12 (0.0%)	87 (0.1%)		
Number of prevalent ADGs at one year before death, mean (SD)	8.4 (4.6)	8.4 (4.6)	8.6 (4.6)	8.5 (4.6)	8.3 (4.7)	<0.001	η^2^ = 0.001
Quartiles of prevalent ADGs at one-year before death, n (%)						<0.001	V = 0.015
0–4	120,367 (30.1%)	30,413 (30.4%)	28,531 (28.5%)	29,720 (29.7%)	31,703 (31.7%)		
5–7	87,685 (21.9%)	22,113 (22.1%)	22,017 (22.0%)	21,936 (21.9%)	21,619 (21.6%)		
8–11	109,172 (27.3%)	27,227 (27.2%)	27,927 (27.9%)	27,609 (27.6%)	26,409 (26.4%)		
12+	83,275 (20.8%)	20,372 (20.4%)	21,650 (21.6%)	20,877 (20.9%)	20,376 (20.4%)		
Received active cancer treatment within the last year of life, n (%)	114,468 (28.6%)	26,556 (26.5%)	28,645 (28.6%)	29,907 (29.9%)	29,360 (29.3%)	<0.001	V = 0.028
Moved within the last year of life, n (%)	62,536 (15.6%)	13,254 (13.2%)	14,382 (14.4%)	15,402 (15.4%)	19,498 (19.5%)	<0.001	V = 0.065

km = kilometer; SD = standard deviation; η^2^ = eta-squared; V = Cramer’s V; ADGs = Johns’ Hopkins adjusted diagnostic groups.

**Fig 1:**
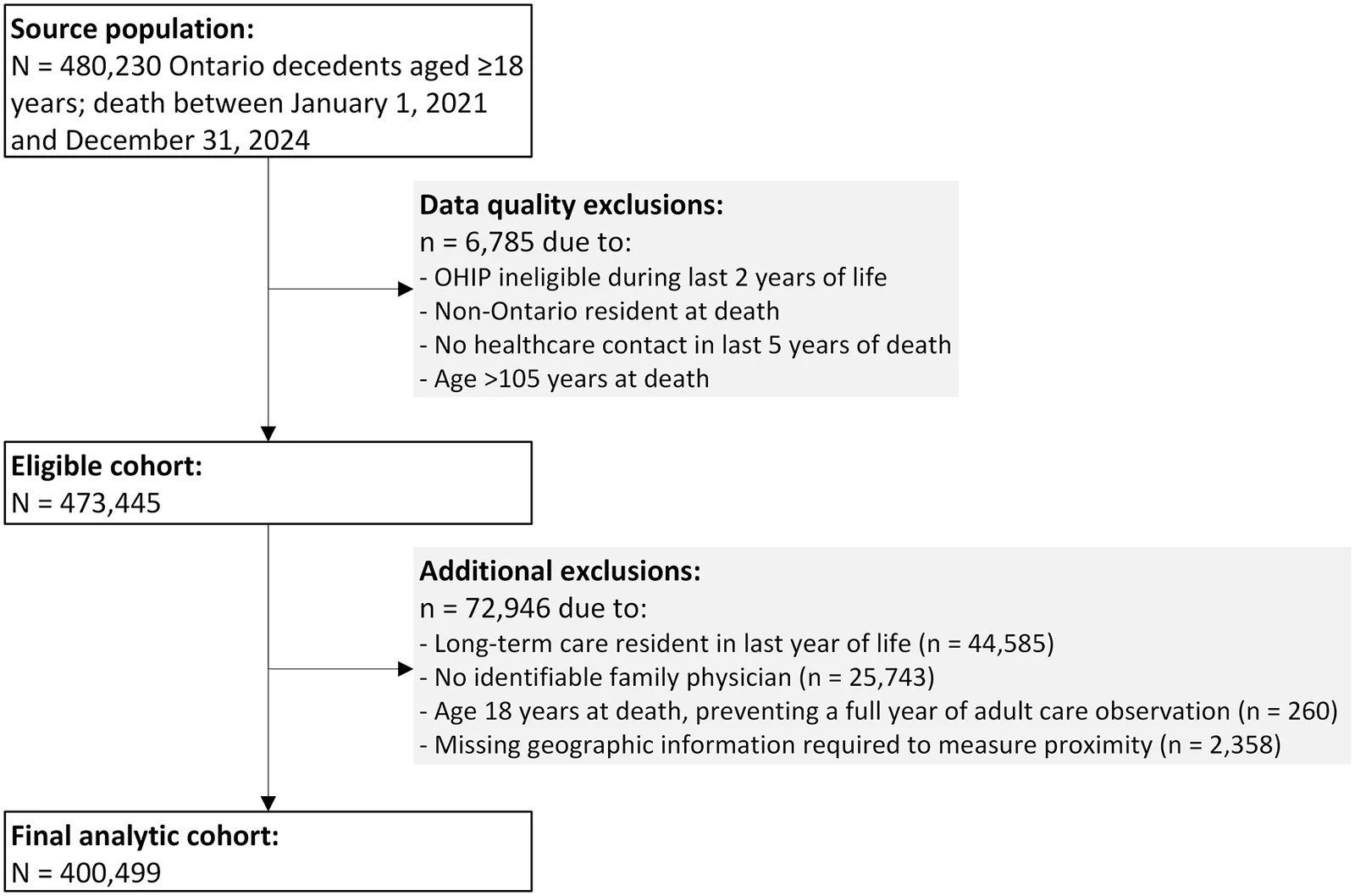
Cohort selection flowchart for adults aged ≥19 years who died in Ontario (2021–2024), showing exclusions and final study population for geographic proximity analysis.

Patients were categorized into quartiles of driving distance to their rostered family physician: Q1(≤ 2.0 km; n = 100,125), Q2 (2.1–5.3 km; n = 100,125), Q3 (5.4–14.2 km; n = 100,142), and Q4 (> 14.2 km; n = 100,107). Differences across quartiles were significant (p < 0.001) due to the large sample size; therefore, effect sizes were used to interpret differences. Rurality status had the largest variation across distance quartiles, ranging as low as 2.8% living at a rural address in Q2 to 24.1% in Q4 (V = 0.239), representing a moderate association. Next, neighbourhood income also varied, with the lowest-income neighbourhoods most common in Q1 (35.6%) compared to Q3 (20.9%) and Q4 (22.1%) (V = 0.093), although representing a smaller effect. Age differed modestly across the quartiles, however, patients living farthest away tended to be younger (Q1: 77.6 (14.2) years; Q4: 72.9 (16.4); η^2^ = 0.015). Sex differences were minimal, with slightly more males in Q4 (54.6%) compared to Q1 (50.6%) (V = 0.032). Fifth, residential mobility during the last year of life was more common among patients in higher distance quartiles (Q1 = 13.2% vs. Q4 = 19.5%; V = 0.065), although the magnitude of this difference was small. Lastly, comorbidity burden (η^2^ = 0.001) and active cancer treatment status (V = 0.028) both demonstrated small variation across distance quartiles.

Measures of continuity varied across quartiles of driving distance to the patient’s family physician (Table 2), although the magnitude of differences were small. The mean UPC index decreased with increasing distance, from 0.35 (0.31) in Q1 to 0.28 (0.31) in Q4 (η^2^ = 0.006) – representing a small effect. The BB index remained stable across quartiles, ranging from 0.64 (0.29) in Q1 to 0.63 (0.30) in Q4 (η^2^ = 0.001), while the RV Index also demonstrated negligible differences (η^2^ < 0.001). Outpatient visit patterns demonstrated fairly small differences across distance quartiles: the mean number of visits was slightly higher in Q2 (17.9 [16.8]) and Q3 (17.7 [16.8]) compared to Q1 (16.2 [16.1]) and Q4 (15.9 [16.3]), respectively (η^2^ = 0.003). Visits with the rostered family physician marginally decreased with greater distance (Q1: 4.8 vs. Q4: 3.8; η^2^ = 0.004), whereas specialist visits and the number of unique physicians involved showed minimal variation (η^2^ = 0.002 and 0.004, respectively).

**Table 2:** Continuity of care indices and outpatient care utilization among adults aged ≥19 years who died in Ontario (2021–2024), stratified by quartiles of driving distance to their rostered family physician.

Variable	Overall(n = 400,499)	Q1: <= 2.0 km(n = 100,125)	Q2: 2.1–5.3 km(n = 100,125)	Q3: 5.4–14.2 km(n = 100,142)	Q4: > 14.2 km(n = 100,107)	P	Effect Size
*Continuity of care indices in the last year of life*							
Usual Provider of Care index, mean (SD)	0.32 (0.31)	0.35 (0.31)	0.32 (0.30)	0.31 (0.30)	0.28 (0.31)	<0.001	η^2^ = 0.006
Modified Bice-Boxerman index, mean (SD)	0.63 (0.29)	0.64 (0.29)	0.62 (0.29)	0.62 (0.29)	0.63 (0.30)	<0.001	η^2^ = 0.001
Relative Variance Index, mean (SD)	0.015 (0.029)	0.014 (0.028)	0.015 (0.028)	0.015 (0.029)	0.015 (0.03)	<0.001	η^2^ = 0.000
*Outpatient care utilization in the last year of life*							
Number of visits, mean (SD)	16.9 (16.5)	16.2 (16.1)	17.9 (16.8)	17.7 (16.8)	15.9 (16.3)	<0.001	η^2^ = 0.003
Number of visits with rostered family physician, mean (SD)	4.5 (6.4)	4.8 (6.5)	4.8 (6.7)	4.6 (6.4)	3.8 (6.1)	<0.001	η^2^ = 0.004
Number of visits with a specialist physician, mean (SD)	12.4 (14.6)	11.4 (14.0)	13.1 (14.8)	13.1 (15.0)	12.2 (14.6)	<0.001	η^2^ = 0.002
Number of unique physicians involved, mean (SD)	5.4 (4.2)	5.1 (4.1)	5.7 (4.3)	5.7 (4.3)	5.2 (4.2)	<0.001	η^2^ =0.004

km = kilometer; SD = standard deviation; η^2^ = eta squared.

### Association between geographic proximity and continuity of care in the last year of life

In unadjusted models, greater driving distance to patients’ usual family physician was associated with lower odds of being in a higher UPC and BB index continuity quartile, but not the RV index (Fig 2). Specifically, each one-quartile increase in driving distance was associated with a decrease in the odds of higher continuity for the UPC (cOR: 0.86; 95% CI: 0.86, 0.87) and BB indices (cOR: 0.98; 95% CI: 0.97, 0.99), while no meaningful association was observed for the RV index (cOR: 1.01; 95% CI: 1.00, 1.02). After adjusting for demographic, socioeconomic, and clinical covariates, the direction and magnitude of these associations remained unchanged (cOR (95% CI): 0.88 (0.87, 0.88) for UPC; 0.98 (0.98, 0.99) for BB; and 1.01 (1.01, 1.02) for RV). Although statistically significant, the magnitude of these associations was modest, consistent with the small effect sizes observed in our descriptive analyses. Additionally, the covariates contributed minimal confounding to the association between geographic proximity and end-of-life continuity of care.

**Fig 2:**
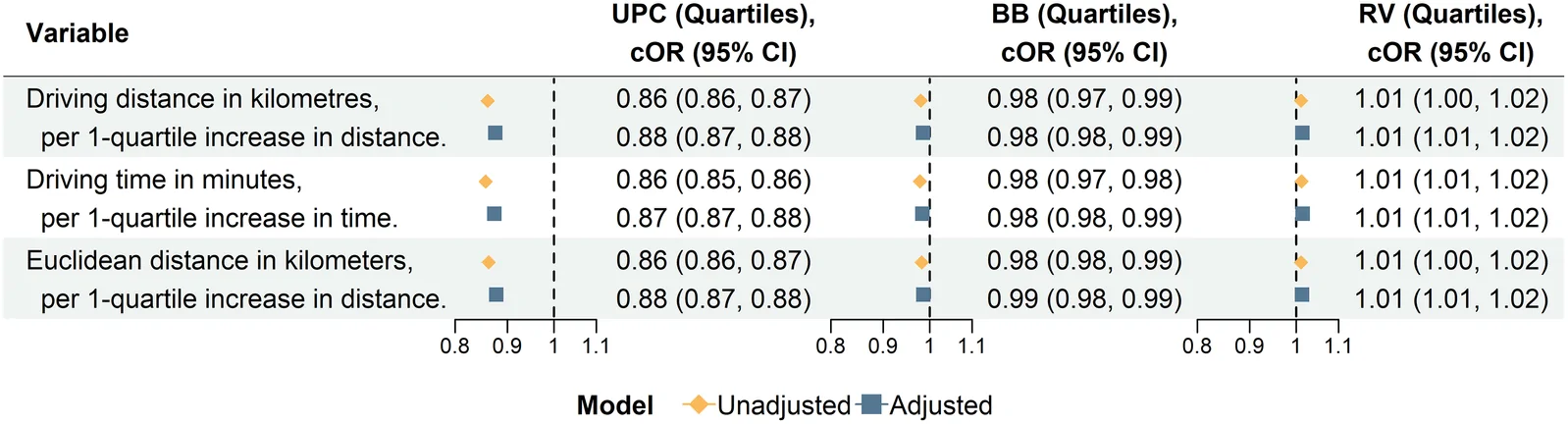
Associations between patients’ geographic proximity to their family physicians and their outpatient continuity of care in the last year of life among adults ≥19 years who died in Ontario (2021–2024).

#### Performance of sensitivity models.

The sensitivity analyses using alternate measures of geographic proximity to family physicians – driving time in minutes and Euclidean distance in kilometers, each modeled in quartiles – yielded nearly identical results to the primary analyses (Fig 2). Additionally, model fit statistics were comparable across specifications, with driving time demonstrating marginally improved model fit. These findings suggest that the observed associations between geographic proximity and end-of-life continuity of care are robust regardless of different operational definitions of geographic proximity (S3 Appendix).

## Discussion

In this population-level study of adults who died in Ontario, Canada from 2021 to 2024, we found that greater geographic distance between patients and their rostered family physicians was associated with modest reductions in outpatient relational continuity during the last year of life. Specifically, patients residing further from their family physicians had lower concentrations of outpatient visits with their family physicians (i.e., lower UPC index scores) and slightly greater dispersion of outpatient care across multiple specialties (i.e., lower BB index scores). However, geographic proximity was not associated with the regularity of family physician visits (i.e., RV index scores), suggesting that while distance influences *who* patients see, it does not affect *how consistently* they seek or receive follow-up care. This finding reflects the continued need for multispecialty care at the end of life, which may be less sensitive to geographic proximity than access to one’s usual care provider.

Our findings also align with Gupta et al.’s population-based cross-sectional study that examined geographic distance among nearly 10 million Ontarians and identified that greater distance was associated with decreased primary care use, and reduced continuity with their family physician [16]. However, Gupta et al.’s study population included a much younger general population (i.e., mean age of ~44 compared to mean age of 76). In comparison, the associations observed in our study were smaller in magnitude, which may reflect differences population characteristics. Regardless, our results expand on Gupta et al.’s findings by measuring the effect of geographic proximity on relational continuity among a high-need end-of-life population. Despite differences in study populations and exposure and outcome definitions, both studies reinforce that geographic proximity is an important contributor to continuity across the care continuum – from chronic disease management to end-of-life care. This is an important finding given the associated benefits of higher usual provider continuity of care on acute care utilization nearing the end of life [30–32]. Although the magnitude of the associations was relatively small, relational continuity of care has been consistently associated with acute care utilization during the end of life. Therefore, even modest reductions in continuity may have meaningful impacts across population levels. As a result, geographic proximity to care providers should be considered as a potential structural determinant of relational continuity along other patient- and system-level factors.

Several mechanisms may explain why distance reduces relational continuity near the end of life. Greater distances may introduce logistical barriers, including transportation challenges (i.e., not having access to personal automotive vehicles or not having driving licenses), caregiver availability (i.e., to drive and attend appointments with patients), mobility limitations, and financial constraints, which are all documented contributors to reduced access and fragmented care [33]. These barriers may cause patients to rely on alternative geographically proximal services – such as urgent care or emergency departments – contributing to care dispersion, contributing to decreased continuity with their family physician and increased care provision from specialist physicians. However, these mechanisms should be interpreted cautiously as health administrative data does not capture the full complexity of patient decision-making. Lastly, patients often receive multispecialty care near the end-of-life which may dilute their relational continuity regardless of their proximity to their care providers [34–37]. As a result, the lack of association between proximity and outpatient visit regularity with family physicians may reflect the unpredictable nature of end-of-life illness trajectories [38], where visit timing may be driven by acute health episodes instead of geographic convenience.

Together, these findings reinforce our protocol’s hypothesis that physical access may be a structural determinant of continuity and care quality. Therefore, system-level strategies may need to address geographic barriers to family medicine at the end of life. First, increasing the number of practicing family physicians in the workforce is vital [39,40], as shortages in primary care workforce capacity limit access to continuous care and contribute to fragmented care across the health system whilst potentially leading to larger catchment areas and greater travel distances – especially in more rural regions. Currently, the provincial government is expanding medical school training capacity to produce more family physicians, extending the Ontario Learn and Stay Grant to support those entering family medicine, and streamlining pathways for internationally trained doctors through the Practice Ready Ontario program [41]. By increasing the number of practising family physicians, these initiatives may help reduce the geographic burden patients face by improving the availability of care closer to where they live. Assuming adequate family physician supply can be achieved, geographically aligned rostering may need to be considered where patients are enrolled with family physicians within the same geographic region or within a shorter travel distance. For individuals living farther away from their existing physicians, strengthening virtual-care pathways, and ensuring appropriate remuneration for virtual encounters may improve accessibility and reduce distance-related continuity decreases. Increasing evidence suggests that virtual modalities can effectively support primary care services such as triage, counselling, and aspects of chronic disease diagnosis and management, comparably to in-person care [42], while improving access to geographically dispersed patient populations, decreasing travel burden, and potential cost savings [43].

### Strengths and limitations

This study has several strengths. First, we leveraged population-level health administrative data and a multi-modal travel-distance measure to measure the relationship between geographic proximity and end-of-life continuity of care. Second, because patients’ full residential address information is unavailable at ICES due to privacy concerns, we used a novel population-weighted geographic centroid to approximate patients’ residences within each 6-character postal code boundary instead of using the geometric centroids. This approach mitigates potential measurement error inherent in postal code-based analyses by better reflecting where people live within these boundaries. Because rural postal-code areas may cover larger geographic regions than urban postal codes, residual measurement error may be greater for some rural residents despite the use of population-weighted centroids. However, these centroids were selected to improve geographic precision relative to conventional geometric centroids and the consistency of findings across multiple proximity measures suggests that any remaining misclassification was unlikely to materially affect our conclusions. Lastly, our sensitivity analyses identified that travel time outperformed travel distance and Euclidean distance, demonstrating superior model fit while providing a more interpretable metric of geographic burden.

This study also has limitations. First, health administrative data does not capture important contextual factors such as patient preferences, perceived barriers to access, transportation availability, or caregiver support, all of which may influence healthcare seeking behaviour and continuity. Second, we were unable to measure non‑billable encounters or care delivered by non-physician care providers such as nurse practitioners – which contribute to continuity but would not be reflected in billing data. Lastly, we excluded individuals who resided in long-term care homes during the last year of life because these residents typically receive care within a distinct healthcare delivery model that differs from community-based primary care. In many cases, physician services are provided by long-term care physicians or physicians affiliated with the facility rather than the patient’s historical community-based family physician. Consequently, inclusion of long-term care residents could introduce misclassification of both geographic proximity and relational continuity measures. Therefore, our findings should be interpreted as applying to community-dwelling adults during the last year of life. However, this exclusion may overlook important geographic dynamics when individuals transition to long-term care. In Ontario, long-term care placement wait times can substantially vary, and as a result, placement policies for certain long-term care admissions allow placement within a 70-kilometer radius (and 150-kilometer in Northern Ontario) of the patient’s preferred location when local vacancies are limited [44]. Therefore, patients’ transitioning to long-term care may experience increasing distance to their family physician. As a result, our study may underestimate how end-of-life residential transitions can further exacerbate geographic family physician separation and affect continuity.

## Conclusion

In brief, geographic proximity to one’s family physician is associated with differences in relational continuity of care during the last year of life. Patients who live farther experience more fragmented outpatient care and lower concentration of visits with their rostered family physician, highlighting the importance of incorporating geographic accessibility into primary care rostering, virtual-care strategies, and end-of-life service planning. Future research should examine how proximity to family physicians’ shapes other important end-of-life healthcare outcomes, such as acute care utilization, accessing palliative homecare services, and location of death, to fully understand how geographic burden influences care patterns across the final stages of life.

## Supporting information

S1 Appendix Description of ICES health administrative data used in the study. (DOCX)

S2 Appendix List of Ontario Health Insurance Plan physician specialties excluded from continuity calculations. (DOCX)

S3 Appendix Goodness of fit for continuity of care models with different distance-based exposure definitions. (DOCX)
