# Supplementary material for: Measuring geographic proximity to family medicine and continuity of care in the last year of life: A population-level retrospective cohort study using Canadian health administrative data
Source: PLoS One. 2026 Oct 16;21(10):e0360638. doi: 10.1371/journal.pone.0360638 (PMC13674724; doi:10.1371/journal.pone.0360638)
Supplement: Supplementary file 1 [file pone.0360638.s001.docx]

S1 Appendix: Description of ICES health administrative data used in the study.

| **Source** | **Content** | **Years** |
| --- | --- | --- |
| Ontario Health Insurance Plan | Includes all physician billing claims for inpatient and outpatient visits, consultations, and procedures, in Ontario. | 2021 - 2024 |
| Ontario Registered Persons Database | Includes demographics, residence, and vital status for all persons eligible for the Ontario Health Insurance Plan. | 2021 - 2024 |
| Corporate Physician Database | Contains administrative information for physicians practicing in Ontario, including practice location address and registration details. | 2021 - 2024 |
| Client Agency Program Enrolment Dataset | Lists patients registered to a primary care organization and links each patient to a family physician and organization type. | 2021 - 2024 |
| Statistics Canada’s Postal Code Conversion File+ | Links 6-character postal codes to Statistics Canada geographic areas, allowing for linkage to census-level demographic and socioeconomic data. | 2021 |
