# Supplementary material for: Measuring geographic proximity to family medicine and continuity of care in the last year of life: A population-level retrospective cohort study using Canadian health administrative data
Source: PLoS One. 2026 Oct 16;21(10):e0360638. doi: 10.1371/journal.pone.0360638 (PMC13674724; doi:10.1371/journal.pone.0360638)
Supplement: Supplementary file 2 [file pone.0360638.s002.docx]

S2 Appendix: List of Ontario Health Insurance Plan physician specialties excluded from continuity calculations.

1. Anaesthesia (01)
2. Critical Care Medicine (11)
3. Emergency Medicine (12)
4. Genetics (22)
5. Laboratory Medicine (28)
6. Microbiology (29)
7. Clinical Biochemistry (30)
8. Diagnostic Radiology (33)
9. Haematology (61)
10. Nuclear Medicine (63)
