# Supplementary material for: Measuring geographic proximity to family medicine and continuity of care in the last year of life: A population-level retrospective cohort study using Canadian health administrative data
Source: PLoS One. 2026 Oct 16;21(10):e0360638. doi: 10.1371/journal.pone.0360638 (PMC13674724; doi:10.1371/journal.pone.0360638)
Supplement: Supplementary file 3 [file pone.0360638.s003.docx]

S3 Appendix: Goodness‑of‑fit for continuity of care models with different distance‑based exposure definitions.

| **Exposure Definition** | **UPC** | **BB** | **RV** |
| --- | --- | --- | --- |
| Driving distance in kilometers: |  |  |  |
| Log-likelihood | -399660 | -375020 | -302730 |
| Akaike Information Criterion | 799400 | 750100 | 605500 |
| Bayesian Information Criterion | 799500 | 750200 | 605600 |
| Driving distance in minutes: |  |  |  |
| Log-likelihood | -399620 | -375020 | -302730 |
| Akaike Information Criterion | 799300 | 750100 | 605500 |
| Bayesian Information Criterion | 799400 | 750200 | 605600 |
| Euclidean distance in kilometers: |  |  |  |
| Log-likelihood | -399710 | -375020 | -302730 |
| Akaike Information Criterion | 799400 | 750100 | 605500 |
| Bayesian Information Criterion | 799600 | 750200 | 605600 |
| ***UPC = Usual Provider of Care index; BB = modified Bice-Boxerman index; RV = Relative Variance Index*** | | | |
