# Supplementary material for: Measuring geographic proximity to family medicine and continuity of care in the last year of life: A population-level retrospective cohort study using Canadian health administrative data
Source: PLoS One. 2026 Oct 16;21(10):e0360638. doi: 10.1371/journal.pone.0360638 (PMC13674724; doi:10.1371/journal.pone.0360638)
Supplement: Supplementary file 4 [file pone.0360638.s005.docx]

**Response to Reviews**

**Manuscript ID:** PONE-D-26-26316

**Title:** Measuring Geographic Proximity to Family Medicine and Continuity of Care in the Last Year of Life: A Population-Level Retrospective Cohort Study Using Canadian Health Administrative Data

**EDITOR**

*Comment 1. PLOS formatting and file naming*

We thank the editors for reviewing our manuscript and for providing their revisions and constructive feedback. We have reviewed our manuscript submission package to ensure that it meets the formatting and file naming conventions required by PLOS.

*Comment 2. Funding statement and acknowledgements*

We have removed the specific funding statement for the Canadian Institutes of Health Research grant from the acknowledgement statements. However, all other acknowledgments must remain as they are required by the respective manuscripts to conduct and report studies leveraging health administrative data.

*Comment 3. Supporting information titles and captions*

We have added a section at the end of the manuscript reporting the supporting information specifically. Additionally, we have revised the formatting in our manuscript references for the appendices to match the formatting criteria required by PLOS.

*Comment 4. Role of funder statement*

We confirm that the online funding statement should be updated to include the following statement:

"The funders had no role in study design, data collection and analysis, decision to publish, or preparation of the manuscript."

*Comment 5. Data availability statement*

The data used in this study are securely held in coded form at ICES. Data-sharing agreements between ICES and data providers prohibit public release of individual-level data. Therefore, access can only be granted to researchers who meet pre-specified criteria for confidential access through ICES Data & Analytic services (<https://www.ices.on.ca/DAS>). As a result, we request an exemption from PLOS’s public deposition requirement as the data contain personal health information which are subject to legal restrictions that prevent public sharing.

**REVIEWER #1**

*Comment 1. Methodological Interpretation: Clinical Significance of "Modest" Effects*

We thank the reviewer for reviewing our manuscript and for providing thoughtful and constructive feedback. We agree that the manuscript can further emphasize the practical implications of the observed associations between geographic proximity and outpatient relational continuity during the last year of life. Our rationale for describing these associations as "modest" was to distinguish statistical significance from clinically meaningful differences in care delivery. Nevertheless, we agree that further context would improve the interpretation of our findings.

As a result, we have revised the Discussion section to further contextualize the observed associations, including their potential population-level implications and their relevance to other healthcare outcomes associated with continuity of care. We also highlight that continuity has been linked to important end-of-life outcomes, suggesting that even relatively small reductions in continuity may have meaningful implications when considered across large populations.

Revisions:

“Although the magnitude of the associations was relatively small, relational continuity of care has been consistently associated with acute care utilization during the end of life. Therefore, even modest reductions in continuity may have meaningful impacts across population levels. As a result, geographic proximity to care providers should be considered as a potential structural determinant of relational continuity along other patient- and system-level factors.” (Lines 284 – 289)

*Comment 2. Justification and Impact of Long-Term Care Exclusions*

We respectfully disagree that the inclusion of long-term care (LTC) residents would represent a sensitivity analysis of the same relationship being studied in our manuscript.

The objective of this study was to measure the association between geographic proximity to a patient's family physician and outpatient relational continuity among community-dwelling adults during the last year of life. LTC residents receive care within a fundamentally different healthcare delivery model in Ontario, where physician services are predominantly provided by LTC physicians or physicians affiliated with the facility rather than the resident's historical community-based family physician. Consequently, the formally rostered family physician may no longer represent the physician providing ongoing primary care.

Therefore, including LTC residents could misclassify both the exposure and outcome variables. Specifically, patients' residential locations become LTC facilities rather than their prior community residences, while continuity measures may reflect care provided by LTC physicians rather than the community-based family physician whose geographic proximity was measured through rostering data. As a result, inclusion of LTC residents would fundamentally alter the interpretation of both the exposure and continuity measures.

For these reasons, we believe that including LTC residents would not address the manuscript's pre-specified objective and would evaluate a different care-delivery context rather than the relationship of interest. However, we agree that our rationale for this exclusion could be more clearly articulated. We have therefore revised both the cohort description and limitations section to further explain the rationale for excluding LTC residents and to clarify that our findings apply to community-dwelling adults in their last year of life.

Revisions:

“We excluded individuals who lived in long-term care homes because these individuals receive care within a healthcare facility where physician services are mostly provided by the on-site physicians instead of their community-based family physicians.” (Lines 118 – 120).

“Lastly, we excluded individuals who resided in long-term care homes during the last year of life because these residents typically receive care within a distinct healthcare delivery model that differs from community-based primary care. In many cases, physician services are provided by long-term care physicians or physicians affiliated with the facility rather than the patient's historical community-based family physician. Consequently, inclusion of long-term care residents could introduce misclassification of both geographic proximity and relational continuity measures. Therefore, our findings should be interpreted as applying to community-dwelling adults during the last year of life.” (Lines 355 – 363)

*Comment 3. Clarification on Geographic Approximation*

We agree that rural postal code areas are often geographically larger than urban postal code areas and, therefore, residual measurement error associated with centroid-based location approximations may be greater for some rural residents. However, the objective of the current study was not to quantify differential measurement error by rurality status.

Nevertheless, we agree that this possibility warrants additional discussion. Accordingly, we have revised the limitations section to acknowledge that residual measurement error may differ between rural and urban regions despite our use of population-weighted centroids, which were selected to improve geographic accuracy relative to conventional geometric centroids.

Revisions:

“Because rural postal-code areas may cover larger geographic regions than urban postal codes, residual measurement error may be greater for some rural residents despite the use of population-weighted centroids. However, these centroids were selected to improve geographic precision relative to conventional geometric centroids and the consistency of findings across multiple proximity measures suggests that any remaining misclassification was unlikely to materially affect our conclusions.” (Lines 340 – 348)

*Minor Comments*

We have made the following changes to address Reviewer #1's minor comments:

1) Supporting information labels have been updated to comply with PLOS ONE formatting requirements.
2) The Python version has been verified and revised as Python 3.11.14.
3) Web-based references have been updated to include access dates.
4) A statement clarifying the use of complete case analysis and the extent of missing covariate data has been added to the Methods section.

**REVIEWER #2**

*Comment 1. Figure 1*

We thank the reviewer for reviewing our manuscript and for providing their meaningful feedback. Figure 1 was originally reported as a cohort flow diagram, however, we agree that it can be improved upon. Therefore, we have revised the figure format to be clearly report the exclusion criteria applied.

Revisions:

Figure 1 has been reformatted to improve clarity and readability.

**ADDITIONAL EDITORIAL COMMENTS**

Funding Statement:

This study was funded by a grant from the Canadian Institutes of Health Research (CIHR), grant #180345.

Role of Funder Statement:

The funders had no role in study design, data collection and analysis, decision to publish, or preparation of the manuscript.

Data Availability:

The underlying data for this study are held securely in coded form at ICES. Data sharing agreements governing these data prohibit public deposition of individual-level datasets. Researchers who meet the criteria for access to confidential health administrative data may obtain access through ICES Data & Analytic Services (DAS). We respectfully request an exemption from PLOS's public data deposition requirement on the basis of legal and privacy restrictions governing the use of Ontario health administrative data.
