# Supplementary material for: Measuring geographic proximity to family medicine and continuity of care in the last year of life: A population-level retrospective cohort study using Canadian health administrative data
Source: PLoS One. 2026 Oct 16;21(10):e0360638. doi: 10.1371/journal.pone.0360638 (PMC13674724; doi:10.1371/journal.pone.0360638)
Supplement: Peer review history

# Peer review: Decision Letter 0

**DOI:** 10.1371/journal.pone.0360638.r001

**Authors:** Bruno Nunes

1 Sep 2026

Dear Dr. Hafid,

Thank you for submitting your manuscript to PLOS One. After careful consideration, we feel that it has merit but does not fully meet PLOS One’s publication criteria as it currently stands. Therefore, we invite you to submit a revised version of the manuscript that addresses the points raised during the review process.

The manuscript has been reassessed by two reviewers. The comments are appended below. There are minor concerns about the manuscript related to the methods, results, and discussion of the paper that affect the technical soundness of your study and the conclusions of the paper. We request authors to conduct a detailed review of the comments and to provide changes in the text.

Please submit your revised manuscript by Oct 01 2026 11:59PM. If you will need more time than this to complete your revisions, please reply to this message or contact the journal office at plosone@plos.org. When you're ready to submit your revision, log on to https://www.editorialmanager.com/pone/ and select the 'Submissions Needing Revision' folder to locate your manuscript file.

- A letter that responds to each point raised by the academic editor and reviewer(s). You should upload this letter as a separate file labeled 'Response to Reviewers'.
- A marked-up copy of your manuscript that highlights changes made to the original version. You should upload this as a separate file labeled 'Revised Manuscript with Track Changes'.
- An unmarked version of your revised paper without tracked changes. You should upload this as a separate file labeled 'Manuscript'.

If you would like to make changes to your financial disclosure, please include your updated statement in your cover letter. Guidelines for resubmitting your figure files are available below the reviewer comments at the end of this letter.

If applicable, we recommend that you deposit your laboratory protocols in protocols.io to enhance the reproducibility of your results. Protocols.io assigns your protocol its own identifier (DOI) so that it can be cited independently in the future. For instructions see: https://journals.plos.org/plosone/s/submission-guidelines#loc-laboratory-protocols. Additionally, PLOS One offers an option for publishing peer-reviewed Lab Protocol articles, which describe protocols hosted on protocols.io. Read more information on sharing protocols at https://plos.org/protocols?utm_medium=editorial-email&utm_source=authorletters&utm_campaign=protocols.

As the corresponding author, your ORCID iD is verified in the submission system and will appear in the published article. PLOS supports the use of ORCID, and we encourage all coauthors to register for an ORCID iD and use it as well. Please encourage your coauthors to verify their ORCID iD within the submission system before final acceptance, as unverified ORCID iDs will not appear in the published article. *Only*  the individual author can complete the verification step; PLOS staff *cannot*  verify ORCID iDs on behalf of authors.

We look forward to receiving your revised manuscript.

Kind regards,

Bruno Pereira Nunes, Ph.D.

Academic Editor

PLOS One

Journal Requirements:

When submitting your revision, we need you to address these additional requirements.

1. Please ensure that your manuscript meets PLOS ONE's style requirements, including those for file naming. The PLOS ONE style templates can be found at

https://journals.plos.org/plosone/s/file?id=wjVg/PLOSOne_formatting_sample_main_body.pdf and

https://journals.plos.org/plosone/s/file?id=ba62/PLOSOne_formatting_sample_title_authors_affiliations.pdf

2. Thank you for stating the following in the Acknowledgments Section of your manuscript:

"This study was supported by ICES, which is funded by an annual grant from the Ontario Ministry of Health (MOH) and the Ministry of Long-Term Care (MLTC). This study also received funding from the Canadian Institutes of Health Research grant #180345. This

document used data adapted from the Statistics Canada Postal Code OM Conversion File, which is based on data licensed from Canada Post Corporation, and/or data adapted from the Ontario Ministry of Health Postal Code Conversion File, which contains data copied under license from ©Canada Post Corporation and Statistics Canada. Parts of this material are based on data and/or information compiled and provided by CIHI and the Ontario MOH. The analyses, conclusions, opinions, and statements expressed herein are solely those of the authors and do not reflect those of the funding or data sources; no endorsement is intended or should be inferred. This study was funded by a grant from the Canadian Institutes of Health Research project #180345."

We note that you have provided additional information within the Acknowledgements Section that is not currently declared in your Funding Statement. Please note that funding information should not appear in the Acknowledgments section or other areas of your manuscript. We will only publish funding information present in the Funding Statement section of the online submission form.

Please remove any funding-related text from the manuscript and let us know how you would like to update your Funding Statement. Currently, your Funding Statement reads as follows:

"This study was funded by a grant from the Canadian Institutes of Health Research project #180345."

Please include your amended statements within your cover letter; we will change the online submission form on your behalf.

3. Please include captions for your Supporting Information files at the end of your manuscript, and update any in-text citations to match accordingly. Please see our Supporting Information guidelines for more information: http://journals.plos.org/plosone/s/supporting-information.

4. Thank you for stating the following financial disclosure:

"This study was funded by a grant from the Canadian Institutes of Health Research project #180345."

Please state what role the funders took in the study.  If the funders had no role, please state: "The funders had no role in study design, data collection and analysis, decision to publish, or preparation of the manuscript."

If this statement is not correct you must amend it as needed.

Please include this amended Role of Funder statement in your cover letter; we will change the online submission form on your behalf.

5. In the online submission form, you indicated that "The data set from this study is held securely in coded form at ICES. While data sharing agreements prohibit ICES from making the data set publicly available, access may be granted to those who meet prespecified criteria for confidential access, available at www.ices.on.ca/DAS. The full data set creation plan and underlying analytic code are available from the authors upon request, understanding that the computer programs may rely upon coding templates or macros that are unique to ICES and are therefore either inaccessible or may require modification."

All PLOS journals now require all data underlying the findings described in their manuscript to be freely available to other researchers, either 1. In a public repository, 2. Within the manuscript itself, or 3. Uploaded as supplementary information.

This policy applies to all data except where public deposition would breach compliance with the protocol approved by your research ethics board. If your data cannot be made publicly available for ethical or legal reasons (e.g., public availability would compromise patient privacy), please explain your reasons on resubmission and your exemption request will be escalated for approval.

6. If the reviewer comments include a recommendation to cite specific previously published works, please review and evaluate these publications to determine whether they are relevant and should be cited. There is no requirement to cite these works unless the editor has indicated otherwise.

7. Please review your reference list to ensure that it is complete and correct. If you have cited papers that have been retracted, please include the rationale for doing so in the manuscript text, or remove these references and replace them with relevant current references. Any changes to the reference list should be mentioned in the rebuttal letter that accompanies your revised manuscript. If you need to cite a retracted article, indicate the article’s retracted status in the References list and also include a citation and full reference for the retraction notice.

[Note: HTML markup is below. Please do not edit.]

Reviewers' comments:

Reviewer's Responses to Questions

**Comments to the Author**

1. Is the manuscript technically sound, and do the data support the conclusions?

Reviewer #1: Yes

Reviewer #2: Yes

**********

2. Has the statistical analysis been performed appropriately and rigorously?

Reviewer #1: Yes

Reviewer #2: Yes

**********

3. Have the authors made all data underlying the findings in their manuscript fully available?

The PLOS Data policy

Reviewer #1: No

Reviewer #2: No

**********

4. Is the manuscript presented in an intelligible fashion and written in standard English?

Reviewer #1: Yes

Reviewer #2: Yes

**********

Reviewer #1: Reviewer Comments to the Author

Manuscript Number: PONE-D-26-26316 Title: Measuring Geographic Proximity to Family Medicine and Continuity of Care in the Last Year of Life: A Population-Level Retrospective Cohort Study Using Canadian Health Administrative Data

General Assessment

This is a well-designed population-level study addressing an important gap in end-of-life (EOL) primary care. The use of linked administrative data from Ontario and the application of established continuity of care (COC) indices provide a rigorous foundation. However, there are several points regarding journal-specific formatting and methodological transparency that should be addressed.

Major Points for Revision

1. Methodological Interpretation: Clinical Significance of "Modest" Effects

The authors repeatedly characterize the associations (e.g., cOR of 0.88 for the UPC index) as "modest". While the large sample size (n=400,499) ensures statistical significance (p < 0.001), the clinical or policy-level impact of a 12% reduction in the odds of higher continuity per quartile increase in distance needs more explicit discussion. Please expand the Discussion to explain what these "modest" reductions mean for a patient’s actual care experience or the likelihood of an emergency department visit, perhaps by referencing your previous work or related literature.

2. Justification and Impact of Long-Term Care (LTC) Exclusions

The study excludes 44,585 individuals (9.3% of the initial dataset) who resided in LTC homes. While the rationale (on-site physician care) is sound, the authors admit this may underestimate the geographic burden during EOL residential transitions. Please provide a brief sensitivity analysis or a more detailed descriptive comparison of the LTC-excluded group versus the included cohort (e.g., age, income) to ensure their exclusion does not introduce significant selection bias regarding geographic access.

3. Clarification on Geographic Approximation

The use of "population-weighted centroids" to approximate residences is a strength for protecting privacy. However, the accuracy of this method can vary significantly between dense urban areas and large, sparsely populated rural postal codes. Please clarify if the "measurement error" noted in the limitations (line 323) was found to differ systematically by rurality status, as this could impact the findings for the highest distance quartile (Q4), which contains the highest proportion of rural residents (24.1%).

Minor Points/Technical Corrections

1. PLOS ONE Formatting: Nomenclature of Supporting Information

The manuscript refers to supplemental materials as "Appendix 1, 2, 3" and a "RECORD Checklist" as "S1 File". To comply with PLOS ONE style, all supporting information should be labeled consistently as "S1 Appendix," "S2 Appendix," etc., and cited as such in the text.

2. Software Version Verification

In the Statistical Analysis section, the authors cite "Python (Version 3.14.0)" Please verify the version number. As of mid-2024, Python 3.14 was in early development/alpha stages. Ensure this is not a typo for an earlier stable version (e.g., 3.11 or 3.12).

3. Reference Formatting

Several references for online tools and reports (e.g., References 24, 25, 27, 41) lack consistent "Accessed [Date]" information. Please ensure all web-based references include the date they were last accessed to meet journal standards.

4. Figure and Table Alignment

In Table 1, "missing" income data is noted for 123 individuals. Briefly mention in the Methods how missing data for covariates (if any beyond income) was handled (e.g., complete case analysis).

Reviewer #2: Overall, I found this to be a well-designed, clearly written, and methodologically rigorous population-based study addressing an interesting and clinically relevant question. The study design and analytic methods are appropriate for the research objectives, and the use of multiple measures of relational continuity and sensitivity analyses using alternative measures of geographic proximity further strengthen the study. The manuscript is also well organized and the findings are presented clearly.

I have only a minor comment. Figure 1 was somewhat difficult to interpret on initial review. I recommend that the authors consider revising the figure, i.e., consider a study flow diagram, to more clearly communicate the information being presented.

**********

what does this mean?). If published, this will include your full peer review and any attached files.

If you choose “no”, your identity will remain anonymous but your review may still be made public.

**Do you want your identity to be public for this peer review?**  For information about this choice, including consent withdrawal, please see our Privacy Policy

Reviewer #1: No

Reviewer #2: No

**********

[NOTE: If reviewer comments were submitted as an attachment file, they will be attached to this email and accessible via the submission site. Please log into your account, locate the manuscript record, and check for the action link "View Attachments". If this link does not appear, there are no attachment files.]

To ensure your figures meet our technical requirements, please review our figure guidelines: https://journals.plos.org/plosone/s/figures

You may also use PLOS’s free figure tool, NAAS, to help you prepare publication quality figures: https://journals.plos.org/plosone/s/figures#loc-tools-for-figure-preparation.

NAAS will assess whether your figures meet our technical requirements by comparing each figure against our figure specifications.

# Author comment: Author response to Decision Letter 1

**DOI:** 10.1371/journal.pone.0360638.r002

18 Sep 2026

Please see the attached “Response to Reviewers” document for our detailed point-by-point responses to all editor and reviewer comments. We have revised the manuscript accordingly and believe that all concerns have been addressed.

Attachment Submitted filename: Response to Reviews 2026-09-17.docx

# Peer review: Decision Letter 1

**DOI:** 10.1371/journal.pone.0360638.r003

**Authors:** Bruno Nunes

30 Sep 2026

Measuring Geographic Proximity to Family Medicine and Continuity of Care in the Last Year of Life: A Population-Level Retrospective Cohort Study Using Canadian Health Administrative Data

PONE-D-26-26316R1

Dear Dr. Hafid,

We’re pleased to inform you that your manuscript has been judged scientifically suitable for publication and will be formally accepted for publication once it meets all outstanding technical requirements.

Within one week, you’ll receive an e-mail detailing the required amendments. When these have been addressed, you’ll receive a formal acceptance letter and your manuscript will be scheduled for publication.

An invoice will be generated when your article is formally accepted. Please note, if your institution has a publishing partnership with PLOS and your article meets the relevant criteria, all or part of your publication costs will be covered. Please make sure your user information is up-to-date by logging into Editorial Manager at Editorial Manager® and clicking the ‘Update My Information' link at the top of the page. For questions related to billing, please contact billing support.

If your institution or institutions have a press office, please notify them about your upcoming paper to help maximize its impact. If they’ll be preparing press materials, please inform our press team as soon as possible -- no later than 48 hours after receiving the formal acceptance. Your manuscript will remain under strict press embargo until 2 pm Eastern Time on the date of publication. For more information, please contact onepress@plos.org.

Kind regards,

Bruno Pereira Nunes, Ph.D.

Academic Editor

PLOS One

Additional Editor Comments (optional):

Reviewers' comments:

Reviewer's Responses to Questions

**Comments to the Author**

Reviewer #1: All comments have been addressed

**********

2. Is the manuscript technically sound, and do the data support the conclusions?

Reviewer #1: Yes

**********

3. Has the statistical analysis been performed appropriately and rigorously?

Reviewer #1: Yes

**********

4. Have the authors made all data underlying the findings in their manuscript fully available?

The PLOS Data policy

Reviewer #1: No

**********

5. Is the manuscript presented in an intelligible fashion and written in standard English?

Reviewer #1: Yes

**********

Reviewer #1: I have no additional comments for the authors. The manuscript has been evaluated based on the questions above, and I have no further concerns regarding its content, methodology, ethical considerations, or publication practices.

**********

what does this mean?). If published, this will include your full peer review and any attached files.

If you choose “no”, your identity will remain anonymous but your review may still be made public.

**Do you want your identity to be public for this peer review?**  For information about this choice, including consent withdrawal, please see our Privacy Policy

Reviewer #1: **Yes:**  IYN-HYANG LEE

**********

# Editor report: Acceptance letter

**DOI:** 10.1371/journal.pone.0360638.r004

**Authors:** Bruno Nunes

PONE-D-26-26316R1

PLOS One

Dear Dr. Hafid,

I'm pleased to inform you that your manuscript has been deemed suitable for publication in PLOS One. Congratulations! Your manuscript is now being handed over to our production team.

At this stage, our production department will prepare your paper for publication. This includes ensuring the following:

* All references, tables, and figures are properly cited

* All relevant supporting information is included in the manuscript submission,

* There are no issues that prevent the paper from being properly typeset

You will receive further instructions from the production team, including instructions on how to review your proof when it is ready. Please keep in mind that we are working through a large volume of accepted articles, so please give us a few days to review your paper and let you know the next and final steps.

Lastly, if your institution or institutions have a press office, please let them know about your upcoming paper now to help maximize its impact. If they'll be preparing press materials, please inform our press team within the next 48 hours. Your manuscript will remain under strict press embargo until 2 pm Eastern Time on the date of publication. For more information, please contact onepress@plos.org.

You will receive an invoice from PLOS for your publication fee after your manuscript has reached the completed accept phase. If you receive an email requesting payment before acceptance or for any other service, this may be a phishing scheme. Learn how to identify phishing emails and protect your accounts at https://explore.plos.org/phishing.

If we can help with anything else, please email us at customercare@plos.org.

Thank you for submitting your work to PLOS One and supporting open access.

Kind regards,

PLOS One Editorial Office Staff

on behalf of

Dr. Bruno Pereira Nunes

Academic Editor

PLOS One
